# Enhanced ascomycin production in *Streptomyces hygroscopicus* var. *ascomyceticus* by employing polyhydroxybutyrate as an intracellular carbon reservoir and optimizing carbon addition

**DOI:** 10.1186/s12934-021-01561-y

**Published:** 2021-03-17

**Authors:** Pan Wang, Ying Yin, Xin Wang, Jianping Wen

**Affiliations:** 1 Key Laboratory of Systems Bioengineering (Ministry of Education), Tianjin University, Tianjin, China; 2 SynBio Research Platform, Collaborative Innovation Center of Chemical Science and Engineering (Tianjin), School of Chemical Engineering and Technology, Tianjin University, Tianjin, China

**Keywords:** Ascomycin, Carbon reservoir, NADH, Polyhydroxybutyrate, Transcriptomics

## Abstract

**Background:**

Ascomycin is a multifunctional antibiotic produced by *Streptomyces hygroscopicus* var. *ascomyceticus*. As a secondary metabolite, the production of ascomycin is often limited by the shortage of precursors during the late fermentation phase. Polyhydroxybutyrate is an intracellular polymer accumulated by prokaryotic microorganisms. Developing polyhydroxybutyrate as an intracellular carbon reservoir for precursor synthesis is of great significance to improve the yield of ascomycin.

**Results:**

The fermentation characteristics of the parent strain *S. hygroscopicus* var. *ascomyceticus* FS35 showed that the accumulation and decomposition of polyhydroxybutyrate was respectively correlated with cell growth and ascomycin production. The co-overexpression of the exogenous polyhydroxybutyrate synthesis gene *phaC* and native polyhydroxybutyrate decomposition gene *fkbU* increased both the biomass and ascomycin yield. Comparative transcriptional analysis showed that the storage of polyhydroxybutyrate during the exponential phase accelerated biosynthesis processes by stimulating the utilization of carbon sources, while the decomposition of polyhydroxybutyrate during the stationary phase increased the biosynthesis of ascomycin precursors by enhancing the metabolic flux through primary pathways. The comparative analysis of cofactor concentrations confirmed that the biosynthesis of polyhydroxybutyrate depended on the supply of NADH. At low sugar concentrations found in the late exponential phase, the optimization of carbon source addition further strengthened the polyhydroxybutyrate metabolism by increasing the total concentration of cofactors. Finally, in the fermentation medium with 22 g/L starch and 52 g/L dextrin, the ascomycin yield of the co-overexpression strain was increased to 626.30 mg/L, which was 2.11-fold higher than that of the parent strain in the initial medium (296.29 mg/L).

**Conclusions:**

Here we report for the first time that polyhydroxybutyrate metabolism is beneficial for cell growth and ascomycin production by acting as an intracellular carbon reservoir, stored as polymers when carbon sources are abundant and depolymerized into monomers for the biosynthesis of precursors when carbon sources are insufficient. The successful application of polyhydroxybutyrate in increasing the output of ascomycin provides a new strategy for improving the yields of other secondary metabolites.

**Supplementary Information:**

The online version contains supplementary material available at 10.1186/s12934-021-01561-y.

## Background

Ascomycin (FK520) is a natural macrocyclic antibiotic produced by *Streptomyces hygroscopicus* var. *ascomyceticus* ATCC 14891 [1]. Because of its specific structure, FK520 exhibits diverse biological and pharmacological activities, including antifungal [2], antimalarial [3], immunosuppressive [4] and anticonvulsive effects [5]. Therefore, FK520 has shown broad application prospect in the clinical treatment of organ transplant rejections, skin diseases and autoimmune diseases [2, 6, 7].

Many recent studies attempted to improve the productivity of engineering strains to meet the growing market demand for FK520. Femtosecond laser irradiation technology was used to increase the yield of FK520 to 240 mg/L, which was 45% higher than that of the parent strain *S. hygroscopicus* var. *ascomyceticus* NT2-11 [8]. Based on ^13^C-metabolic flux analysis, the deletion of *pyc* gene (encoding pyruvate carboxylase, competing for pyruvate with FK520 precursors) and the overexpression of *fkbO* gene (responsible for the biosynthesis of the FK520 starting unit) increased the production of FK520 to 550 mg/L, which was 66.7% higher than that of the parent strain *S. hygroscopicus* var. *ascomyceticus* SA68 [9]. In addition, the co-overexpression of the regulator *fkbR1* and its target gene *fkbE* improved the FK520 yield to 536.7 mg/L, which was 69.9% higher than that of the parent strain *S. hygroscopicus* var. *ascomyceticus* FS35 [7]. However, the supply of carbon sources for the synthesis of FK520 precursors in the late phase of fermentation is still an unresolved bottleneck. The excessive addition of carbon sources into the fermentation medium inhibits the growth of the cells and decreases the yield of target products. For example, the addition of more than 80 g/L soluble starch in the fermentation medium inhibited the accumulation of biomass and the biosynthesis of FK520 by *S. hygroscopicus* ar. *ascomyceticus* SFK-36 [10]. Similarly, the addition of more than 5 g/L glucose into the fermentation medium inhibited the production of rapamycin by *S. hygroscopicus* HD-04 [11]. Therefore, the development and utilization of intracellular carbon reservoirs is of great significance for the synthesis of antibiotics during the late fermentation stages.

Polyhydroxybutyrate (PHB) is a multifunctional natural polymer widespread in prokaryotic species [12, 13]. In vitro, because of its biocompatibility, thermoplasticity and biodegradability, PHB can be used to produce biodegradable plastics, such as disposable packaging goods or medical equipment, which have been considered as a green alternative to traditional petrochemical plastics [14–16]. In vivo, PHB is accumulated in the form of insoluble particles when there is an excess of carbon sources in the environment, and decomposed for reuse when there is no suitable carbon source, so it regarded as an intracellular carbon storage granule [17]. In previous studies, PHB was shown to improve the production of a number of primary metabolites. For example, the introduction of the PHB biosynthesis pathway into *Escherichia coli* QZ1111 improved the output of succinate by increasing the flux of pyruvate into the citrate cycle (TCA) [18]. Similarly, the introduction of the PHB synthesis pathway into *Corynebacterium crenatum* increased the L-arginine yield by regulating the corresponding carbon flows [19]. However, the effect of PHB on antibiotic production has not been reported to date.

In *S. hygroscopicus* var. *ascomyceticus*, FK520 is assembled from 12 precursor molecules [20], nearly half of which (5 molecules of methylmalonyl-CoA and 1 molecule of ethylmalonyl-CoA) can be derived from the ethylmalonyl-CoA pathway (EMCP) [21]. The EMCP is mainly responsible for the assimilation of C_2_-dicarboxylic acids into C_4_ or C_5_-units [22]. In addition, in some *Streptomyces* species, the assimilation products of EMCP supply CoA-ester precursors for the biosynthesis of antibiotics [23]. It is worth noting that the ethylmalonyl-CoA from the EMCP is a specific precursor for FK520, which can be used to construct the core structure of FK520 that distinguishes it from other similar antibiotics [24, 25]. Therefore, maintaining the carbon source supply for EMCP is critical for the biosynthesis of FK520. The carbon flux through the EMCP mainly depends on the supply of 3-hydroxybutyryl-CoA [26], which can also be obtained from the decomposition of PHB. Thus, it is of great significance to explore the influence of PHB on the production of FK520 and develop PHB as an intracellular reservoir of carbon sources for the biosynthesis of FK520.

Previous studies demonstrated a variety of novel approaches for the bioconversion and biotechnological production of antibiotics, including new fed-batch fermentation strategies, innovative precursor supplementation approaches and specific bioreactor designs [27]. In this study, the bioconversion of PHB into biomass and FK520 was verified by a combination of genetic manipulations and systematic transcriptional analysis, confirming the potential of PHB as an intracellular carbon reservoir. The successful application of PHB in improving the production of FK520 provides a new strategy for increasing the yield of secondary metabolites.

## Results

### Correlation between the polyhydroxybutyrate content and ascomycin production

**Fig. 1:**
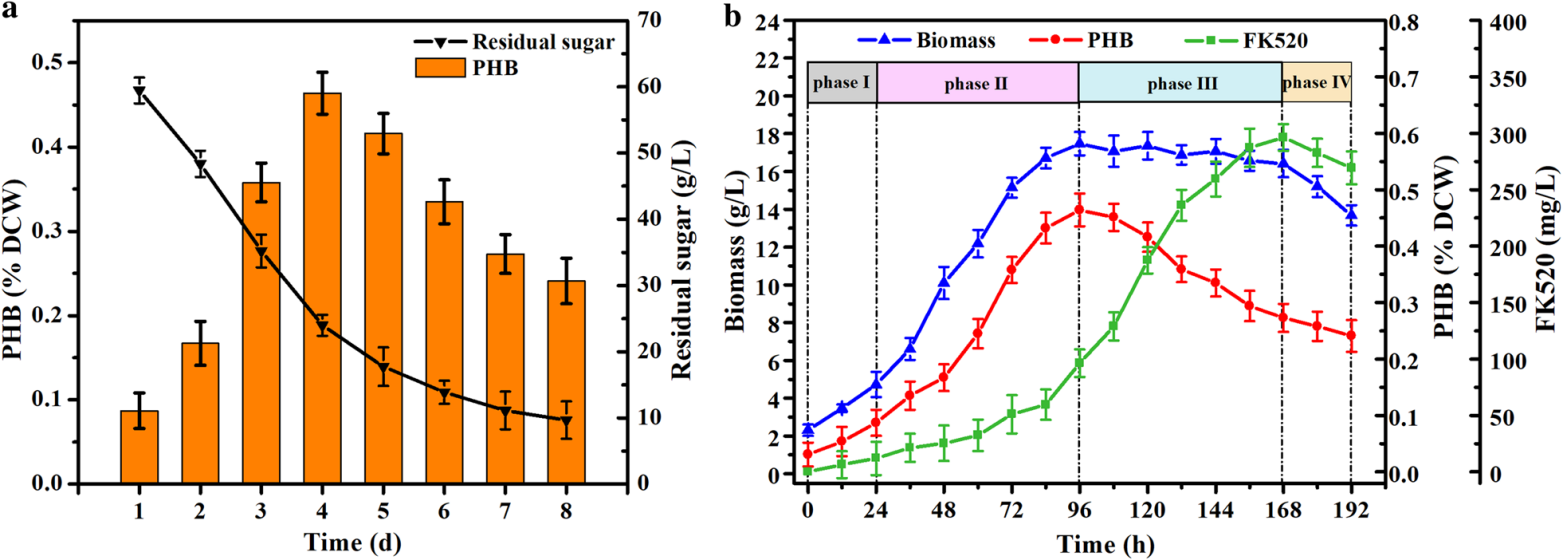
Correlation between polyhydroxybutyrate metabolism, biomass accumulation and ascomycin production. **a** The change trends of polyhydroxybutyrate content and residual sugar in the parent strain FS35. **b** The correlation between the polyhydroxybutyrate content, biomass and ascomycin production in the parent strain FS35. Phase I, lag phase; Phase II, exponential phase; Phase III, stationary phase; Phase IV, decline phase. The data represent the mean values of five independent biological replicates, and the error bars represent the standard deviations

Various prokaryotic microorganisms are known to accumulate PHB [28], but its presence in *S. hygroscopicus* var. *ascomyceticus* was uncertain. Here, the parent strain FS35 was fermented for 8 days to investigate the change trends of the PHB contents and residual sugars during the entire fermentation (Fig. 1a). In strain FS35, the PHB content gradually increased along with the decrease of residual sugars during the first four days, but gradually decreased when residual sugar was already at low level during the last four days. This suggested that PHB was first accumulated and then degraded in *S. hygroscopicus* var. *ascomyceticus*, and the monomers released by the degradation of PHB during the later stage of fermentation might have other uses.

It had been reported that a PHB degradation gene is present in the FK520 gene cluster of *S. hygroscopicus* var. *ascomyceticus* [1]. Thus, in order to assess whether the degradation of PHB was related to the biosynthesis of FK520, more detailed fermentation characteristics of FS35 were recorded and analyzed. As shown in Fig. 1b, when PHB was accumulated rapidly during the exponential phase, the biomass also increased correspondingly. Excitingly, when PHB was decomposed during the stationary phase, the production of FK520 rapidly increased. This indicated that PHB metabolism might be related to both cell growth and FK520 synthesis.

### The promoting effect of polyhydroxybutyrate metabolism on strain growth and ascomycin production

To explore the influence of PHB metabolism on the production of FK520, the native PHB degradation gene *fkbU* was overexpressed in the parent strain FS35 to construct the overexpressed strain OfkbU. However, the FK520 production of strain OfkbU was not significantly improved (Additional file 1: Figure S1). This can be explained by the fact that the amount of PHB synthesized by OfkbU during the exponential phase was not increased, so there was no more PHB available for degradation during the stationary phase. Previous studies reported that PHB synthase and depolymerase are simultaneously expressed in most PHB-accumulating strains, such as *Ralstonia eutropha* H16, but their activities are stringently regulated to avoid ineffective circulation [29, 30]. Therefore, the combined overexpression of the PHB synthesis gene and decomposition gene in strain FS35 was deemed a reasonable approach. The PHB synthesis gene (*phaC*) from *R. eutropha* H16 had been widely studied and used [31]. Therefore, the exogenous PHB synthesis gene (*phaC*) and the native PHB decomposition gene (*fkbU*) were co-overexpressed in the parent strain FS35 to construct the co-overexpression strain OphaCfkbU.

**Fig. 2:**
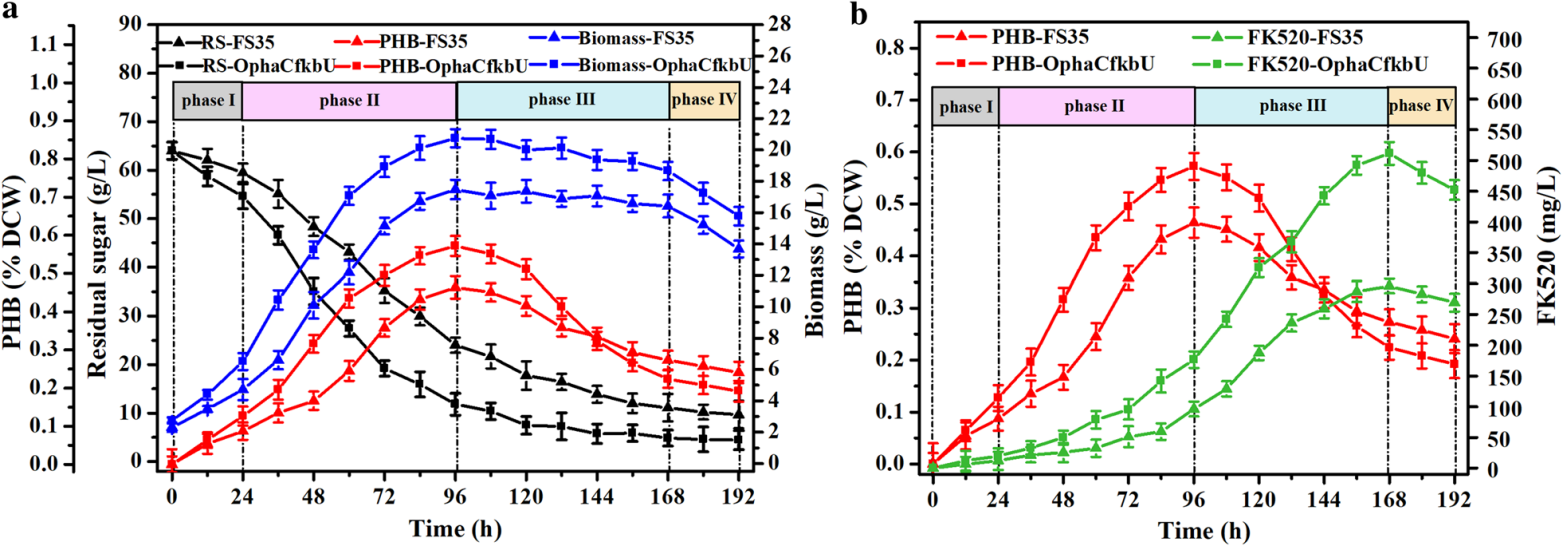
Comparison of fermentation characteristics between the parent strain FS35 and the co-overexpression strain OphaCfkbU. **a** The comparison of residual sugar, biomass and polyhydroxybutyrate content between strains FS35 and OphaCfkbU during the different fermentation phases. *RS* in the legend stands for residual sugar. **b** The comparison of polyhydroxybutyrate content and ascomycin production between strains FS35 and OphaCfkbU during the different fermentation phases. Phase I, lag phase; Phase II, exponential phase; Phase III, stationary phase; Phase IV, decline phase. The data represent the mean values of five independent biological replicates, and the error bars represent the standard deviations

The parent strain FS35 and co-overexpressed strain OphaCfkbU were simultaneously fermented for 192 h to observe the changes of fermentation characteristics caused by the co-overexpression (Figs. 2a, b). Compared to the parent strain FS35, the co-overexpression strain OphaCfkbU consumed more sugar and accumulated more PHB during the exponential phase (Fig. 2a). At the same time, OphaCfkbU also accumulated more biomass during the exponential phase than strain FS35 (Fig. 2a). The thickness of mycelia in the fermentation broth at 96 h and the growth state of mycelia on plates at 20 days showed that the increase of biomass was not only due to increase the accumulation of PHB, but also due to the better growth of strain OphaCfkbU itself (Additional file 1: Figure S2). All of these findings indicated that the biosynthesis of PHB was beneficial for cell growth. As expected, the more abundant supply of PHB in strain OphaCfkbU during the exponential phase ensured more decomposition of PHB during the stationary phase (Fig. 2b). Accordingly, the yield of FK520 in strain OphaCfkbU was also increased to 511.50 mg/L, which was 1.73-fold higher than that of strain FS35 (296.29 mg/L). These results suggested that the accumulation and degradation of PHB was a valuable cycle for the production of FK520.

### Transcriptomic evidence for the stimulating effect of polyhydroxybutyrate on strain growth

**Fig. 3:**
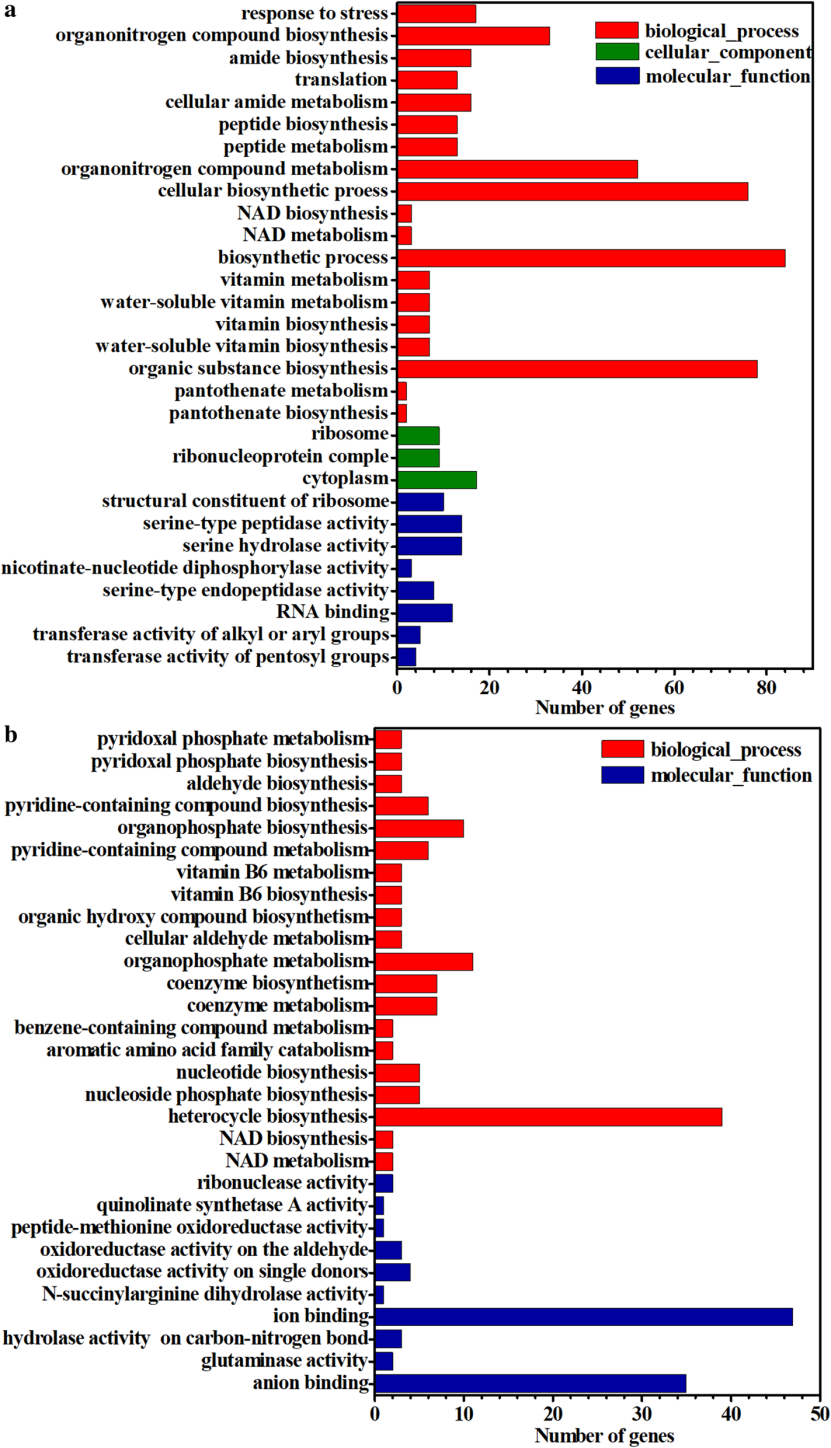
Gene ontology enrichment analysis of differentially expressed genes in the strain OphaCfkbU. **a** The enrichment categories and numbers of the up-regulated genes in strain OphaCfkbU. **b** The enrichment categories and numbers of the down-regulated genes in strain OphaCfkbU. The Y-axes represent different enrichment categories. The X-axes represent the numbers of genes enriched in the corresponding categories, reflecting the degree of enrichment

To provide further evidence for the stimulating effect of PHB metabolism on strain growth, comparative transcriptomic analysis between strain FS35 and OphaCfkbU was carried out using RNA samples drawn at 50 h. Differential expression analysis revealed 285 up and 218 down-regulated genes in strain OphaCfkbU compared to strain FS35 (Additional file 1: Figure S3). These differentially expressed genes were clustered into three categories (biological process, cellular component and molecular function) using gene ontology (GO) enrichment analysis (Figs. 3a, b). The up-regulated genes were mostly related to the biosynthesis and metabolism of cellular molecules (Fig. 3a). The down-regulated genes were mainly involved in the biosynthesis of heterocyclic organics, which is unfavorable for biomass accumulation (Fig. 3b). These results provided evidences for the stimulating effect of PHB on strain growth in both positive and negative aspects.

Furthermore, the significantly up-regulated genes in strain OphaCfkbU were mainly mapped to six metabolic pathways according to the Kyoto encyclopedia of genes and genomes (KEGG) enrichment analysis (Table 1). The expression of genes in the starch and sucrose metabolism was up-regulated 1.16- to 3.08-fold, confirming that the accumulation of PHB promoted the utilization of sugars. The expression of genes in the ABC transport system was up-regulated 1.01- to 4.33-fold, which reflected that PHB also accelerated the transport of sugars and metal ions. In addition, the transcriptional levels of genes in the ribosome biosynthesis pathway increased 1.02- to 5.16-fold, indicating that PHB increased the generation of ribosomes. The transcriptional levels of genes in the pantothenate and coenzyme A biosynthesis pathways increased 1.13- to 1.97- fold, reflecting that PHB also promoted the biosynthesis of coenzyme A and acyl-carrier protein (ACP). Moreover, 1.20- to 2.86-fold higher expression of genes in the nicotinate and nicotinamide metabolism demonstrated that PHB increased the biosynthesis of cofactors (NADH, NAD^+^, NADPH, NADP^+^). Similarly, 1.02- to 5.16-fold higher expression of genes in sulfur metabolism indicated that the synthesis of PHB promoted the synthesis of sulfur-containing compounds. All of these data proved that PHB could stimulate the growth of the strain by promoting the utilization of carbon sources and accelerating the biosynthesis processes.

**Table 1:** Main enriched metabolic pathways of up-regulated genes

Gene no.	KEGG ID	Enzyme name	Log_2_ (change fold)^a^	P value^a^
Starch and sucrose metabolism				
1_2422	SHJG_3718	alpha-glucosidase	3.0752	6.06 × 10^–148^
1_2417	SHJG_3717	alpha-amylase	2.5774	1.81 × 10^–16^
1_3621	SHJG_4827	trehalose-6-phosphate synthase	1.266	2.10 × 10^–59^
1_6067	SHJG_7159	alpha-amylase	1.2268	4.07 × 10^–07^
1_7923	SHJG_1896	beta-glucosidase	1.1619	6.11 × 10^–06^
ABC transporters				
1_1441	SHJG_3980	zinc transport system substrate-binding protein	4.3311	7.75 × 10^–58^
1_2420	SHJG_3720	maltooligosaccharide transport system permease protein	4.2287	8.35 × 10^–41^
1_1444	SHJG_3235	iron complex transport system ATP-binding protein	4.0095	1.55 × 10^–29^
1_2421	SHJG_3721	maltooligosaccharide transport system substrate-binding protein	3.9837	0
1_2419	SHJG_3719	maltooligosaccharide transport system permease protein	3.6265	5.76 × 10^–32^
1_2743	SHJG_3235	iron complex transport system ATP-binding protein	2.9746	4.61 × 10^–57^
1_2741	SHJG_3982	zinc transport system permease protein	2.9356	2.58 × 10^–257^
1_2742	SHJG_8417	iron complex transport system ATP-binding protein	2.9089	1.10 × 10^–83^
1_1279	SHJG_8416	iron complex transport system permease protein	1.8523	5.37 × 10^–08^
1_1280	SHJG_8416	iron complex transport system permease protein	1.7734	8.39 × 10^–07^
1_3705	SHJG_4851	multiple sugar transport system ATP-binding protein	1.518	6.19 × 10^–29^
1_1281	SHJG_8418	iron complex transport system substrate-binding protein	1.4464	1.39 × 10^–23^
1_4985	SHJG_3981	zinc transport system ATP-binding protein	1.0094	8.97 × 10^–11^
Ribosome				
1_1485	SHJG_6688	50S ribosomal protein L32	5.1594	7.62 × 10^–181^
1_7134	SHJG_5164	30S ribosomal protein S18	3.8835	3.44 × 10^–52^
1_7129	SHJG_7368	30S ribosomal protein S14	3.8384	1.82 × 10^–14^
1_7130	SHJG_7369	50S ribosomal protein L28	3.7143	1.33 × 10^–05^
1_2812	SHJG_4055	30S ribosomal protein S20	1.1893	9.08 × 10^–12^
1_5742	SHJG_6711	30S ribosomal protein S16	1.0786	4.86 × 10^–25^
1_6570	SHJG_3036	large subunit ribosomal protein L20	1.0423	1.61 × 10^–10^
1_4696	SHJG_5834	30S ribosomal protein S9	1.0196	8.92 × 10^–25^
Pantothenate and CoA biosynthesis				
1_2437	SHJG_3745	3-methyl-2-oxobutanoate hydroxymethyltransferase	1.9693	5.22 × 10^–56^
1_8015	SHJG_7832	L-aspartate-alpha-decarboxylase	1.7699	4.69 × 10^–07^
1_3771	SHJG_5555	pantoate-beta-alanine ligase	1.6344	1.24 × 10^–05^
1_3768	SHJG_5558	type III pantothenate kinase	1.4657	5.69 × 10^–07^
1_5666	SHJG_6609	acetolactate synthase	1.1284	1.07 × 10^–15^
Nicotinate and nicotinamide metabolism				
1_7327	SHJG_8783	nicotinate phosphoribosyltransferase	2.8566	3.75 × 10^–09^
1_3770	SHJG_5556	L-aspartate oxidase	1.7152	6.70 × 10^–13^
1_7638	SHJG_2643	succinate-semialdehyde dehydrogenase	1.7112	3.27 × 10^–233^
1_3769	SHJG_5557	nicotinate-nucleotide pyrophosphorylase	1.5887	9.02 × 10^–06^
1_3164	SHJG_4395	nicotinamidase	1.1981	5.28 × 10^–18^
Sulfur metabolism				
1_1485	SHJG_6688	50S ribosomal protein L32	5.1594	7.62 × 10^–181^
1_7134	SHJG_5164	30S ribosomal protein S18	3.8835	3.44 × 10^–52^
1_7129	SHJG_7368	30S ribosomal protein S14	3.8384	1.82 × 10^–14^
1_7130	SHJG_7369	50S ribosomal protein L28	3.7143	1.33 × 10^–05^
1_2812	SHJG_4055	30S ribosomal protein S20	1.1893	9.08 × 10^–12^
1_5742	SHJG_6711	30S ribosomal protein S16	1.0786	4.86 × 10^–25^
1_6570	SHJG_3036	large subunit ribosomal protein L20	1.0423	1.61 × 10^–10^
1_4696	SHJG_5834	30S ribosomal protein S9	1.0196	8.92 × 10^–25^

^a^The data was the comparative values between the strain FS35 and strain OphaCfkbU, with the strain FS35 as the control

### The mechanism underlying the promoting effect of polyhydroxybutyrate on ascomycin production

**Fig. 4:**
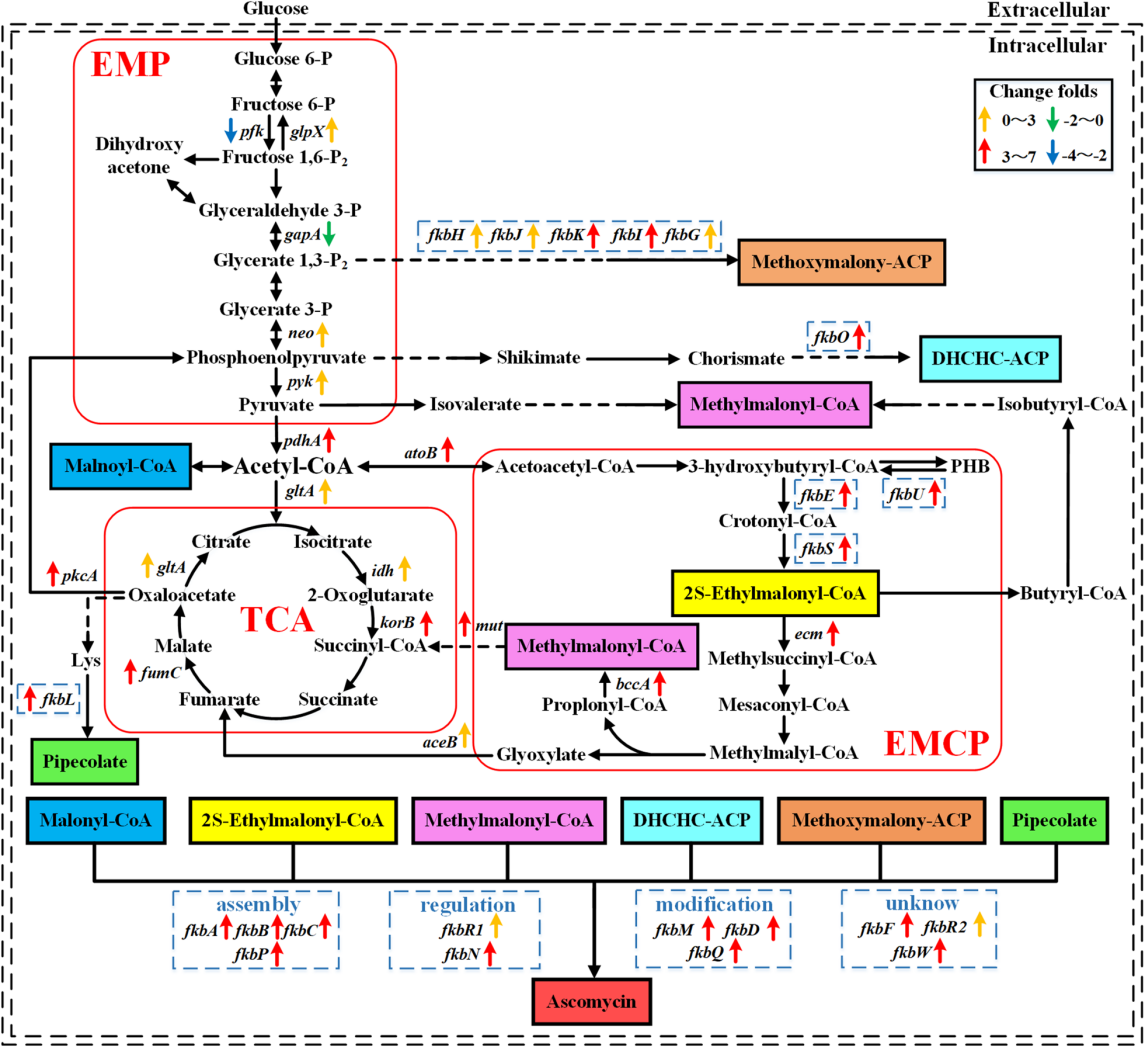
Influence of polyhydroxybutyrate metabolism on ascomycin synthesis. An schematic view of the major metabolic pathways for the synthesis of ascomycin precursors. And the changes in the transcriptional levels of key genes for ascomycin synthesis in strain OphaCfkbU. The transcriptional levels of genes in the parent strain FS35 were defined as 1 and used as the control. The solid lines represent one-step reactions. And the dotted lines represent multi-step reactions. Different colored shaded boxes represent different precursors of ascomycin. The italics represent genes. Blue dotted boxes represent the genes in the gene cluster of ascomycin. The up- and downward facing arrows in different colors represent the different change folds of transcriptional levels as shown in the legend

In order to investigate the mechanism through which PHB depolymerization increases FK520 biosynthesis, transcriptional levels of crucial genes in the primary metabolic pathways and all genes in the FK520 gene cluster were compared between strains FS35 and OphaCfkbU at 112 h (Fig. 4). In strain OphaCfkbU, the transcription of genes in EMCP was 3.90 to 4.25 times higher than in parent strain FS35. This reflected that the degradation of PHB increased the biosynthesis of the precursors ethylmalonyl-CoA and methylmalonyl- CoA by enhancing the metabolic flux through the EMCP. Furthermore, the expression of genes in the TCA and the gene *fkbL* was up-regulated between 1.94 and 5.87 times, indicating that PHB also promoted the biosynthesis of the precursor pipecolate by strengthening the carbon flux through the TCA (Fig. 4). In addition, the transcription of the genes *neo*, *fkbO, fkbH*, *fkbJ*, *fkbK*, *fkbI* and *fkbG* increased 1.83 to 4.77-fold, which showed that PHB also improved the biosynthesis of the precursors methoxymalonyl-ACP and 4,5-dihydroxycyclohex-1-enecarboxylic acid (DHCHC) by regulating the carbon flow through the EMP (Fig. 4). Similarly, the expression of other genes in the FK520 gene cluster, responsible for the assembly, regulation and modification of precursors, was also up-regulated 2.33 to 5.15 times (Fig. 4). This confirmed that PHB metabolism increased the production of FK520 by improving the supply of precursors.

**Fig. 5:**
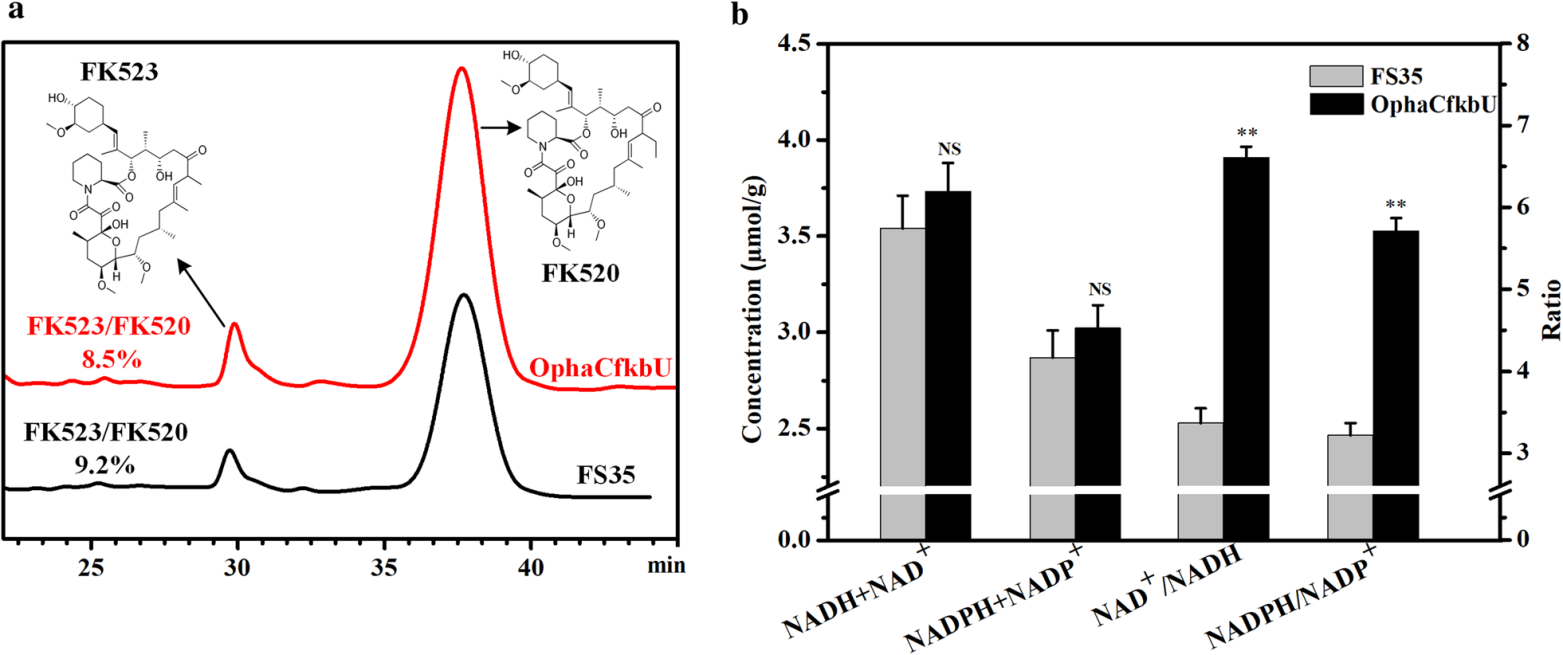
Effect of polyhydroxybutyrate on by-product yield and influence of cofactors on polyhydroxybutyrate biosynthesis. **a** The overlapped chromatograms of ascomycin and by-product in strains FS35 and OphaCfkbU, including the structures and ratios. **b** The comparison of cofactor concentration and ratio between strains FS35 and OphaCfkbU at the end of exponential phase. The data represent the mean values of five independent biological replicates, and the error bars represent the standard deviations. *Asterisks* indicate the significant differences between initial medium and optimized fermentation medium. *P* values were calculated by two-taild Student’s *t* test. Double asterisk indicates *P* < 0.01. *NS* means the difference is not significant

FK523 is the main impurity in the production of FK520, resulting from the assembly of methylmalonyl-CoA onto the C21 position of the macrolide skeleton instead of the specific precursor ethylmalonyl-CoA [32]. To rule out competitive use of these precursors by for by-product synthesis, the yield of FK523 and the FK523/FK520 ratio in the strains FS35 and OphaCfkbU was determined as shown in Fig. 5a. Although the yield of FK523 in the strain OphaCfkbU was higher than in the parent strain FS35, the FK523/FK520 ratio decreased slightly. This indicated that the increase of precursors was mainly beneficial for the synthesis of FK520 but not FK523. It is possible that the degradation of PHB promoted the synthesis of ethylmalonyl-CoA more than methylmalonyl-CoA, which was in agreement with the fold-changes in the expression of *fkbE* (3.98), *fkbS* (4.12), *ecm* (3.35) and *bccA* (3.27). Therefore, the priority supply of carbon from PHB for the specific precursor ethylmalonyl-CoA guaranteed that the increase of FK520 production was not affected by the by-products.

### The influence of cofactor concentrations on polyhydroxybutyrate metabolism

To further develop the value of PHB as an intracellular carbon reservoir, the factors influencing the synthesis of PHB were explored. The conversion of acetoacetyl-CoA into 3-hydroxybutyryl-CoA is an important step for the biosynthesis of PHB. It is catalyzed by the NADH-dependent 3-hydroxybutyryl-CoA dehydrogenase (encoded by *hcd*) or the NAD(P)H-dependent acetoacetyl-CoA reductase (encoded by *phaB*). The NAD(P)H pool and the ratio of NAD(P)H/NAD(P)^+^ could significantly influence the content of PHB [33, 34]. The sequence alignment showed that there is a copy of the *hcd* gene in the genome of *S. hygroscopicus* var. *ascomyceticus* (Additional file 1: Figure S4), but no copy of a *phaB* gene. Thus it was inferred that the synthesis of PHB in *S. hygroscopicus* var. *ascomyceticus* might be influenced by the pool of NADH and the ratio of NADH/NAD ^+^. To test this inference, the concentrations of NADH, NAD^+^, NADPH, NADP^+^ and the ratio of NAD^+^/NADH, NADPH/NADP^+^ in strain FS35 and OphaCfkbU were measured at the end of the exponential phase.

Compared with strain FS35, the total concentration of NADH and NAD^+^ in strain OphaCfkbU did not show a significant increase, but the ratio of NAD^+^/NADH significantly increased at the end of the exponential phase (Fig. 5b). This indicated that in OphaCfkbU, the conversion of NADH to NAD^+^ was greatly improved during the exponential phase. Conversely, although the total concentration of NADPH and NADP^+^ in strain OphaCfkbU also did not increase significantly, the ratio of NADPH/NADP^+^ was significantly higher than in strain FS35 at the end of the exponential phase (Fig. 5b). This suggested that the production rate of NADPH in OphaCfkbU was increased during the exponential phase. Taken together, these results showed that in OphaCfkbU, the increase of PHB biosynthesis during the exponential phase accelerated the consumption of NADH, indicating that the synthesis of PHB depends on the conversion of NADH into NAD^+^. Therefore, increasing the intracellular concentration of NADH might be an effective strategy to further enhance the PHB metabolism.

### Further increase of the ascomycin yield by strengthening the polyhydroxybutyrate metabolism

**Fig. 6:**
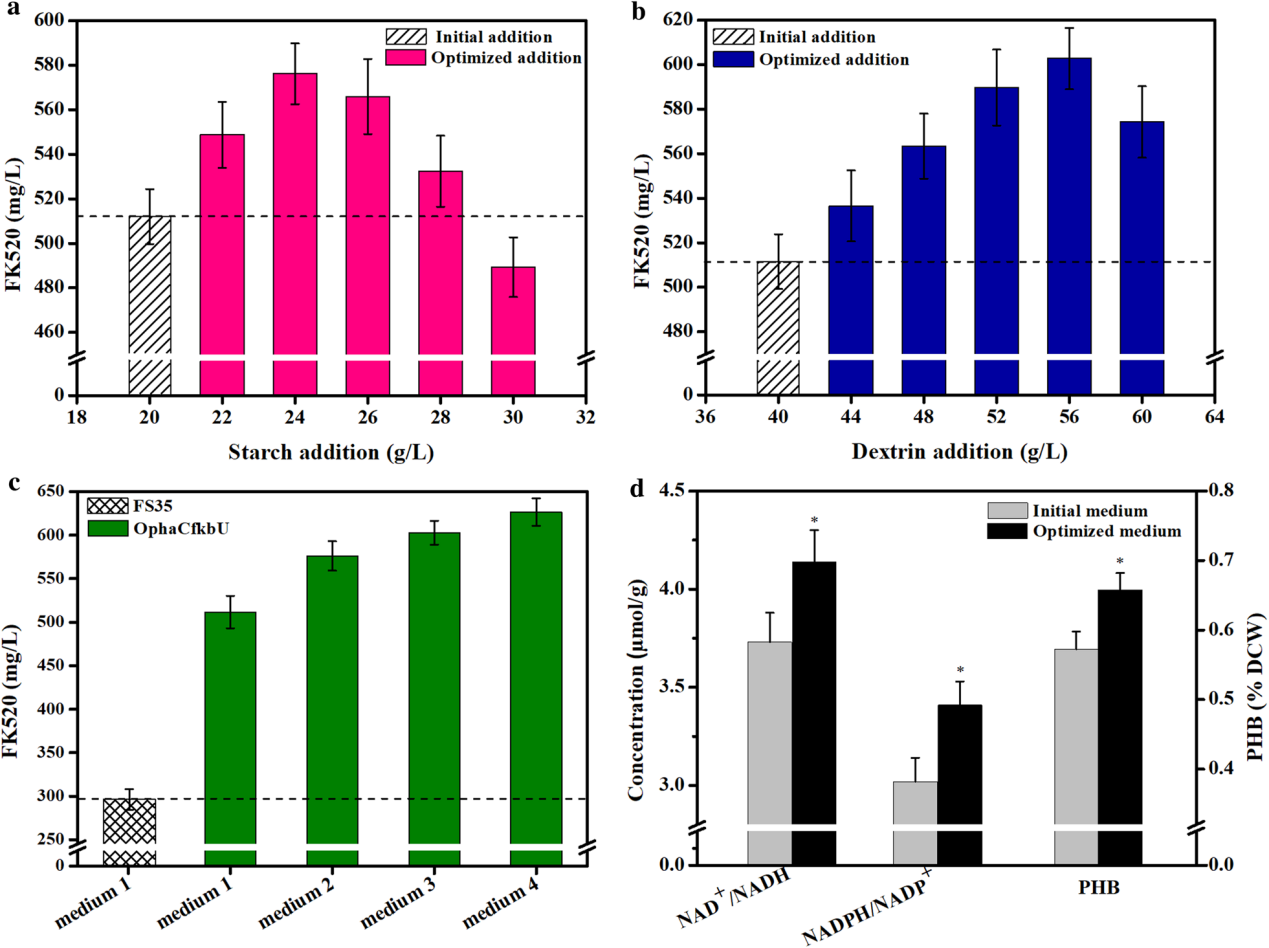
Influence of carbon source addition on the cofactor concentrations, polyhydroxybutyrate content and ascomycin yield. **a** The effect of starch addition on the ascomycin yield of strain OphaCfkbU. **b** The effect of dextrin addition on the ascomycin yield of strain OphaCfkbU. **c** The comparison of ascomycin yield in different strains or different fermentation mediums. Medium 1 respresents the initial fermentation medium. Medium 2 respresents the fermentation medium with optimized starch addtion. Medium 3 respresents the fermentation medium with optimized dextrin addtion. Medium 4 respresents the final optimized fermentation medium. **d** The comparison of cofactor concentration and polyhydroxybutyrate content of strain OphaCfkbU in different fermentation medium at the end of exponential phase. The data represent the mean values of five independent biological replicates, and the error bars represent the standard deviations. *Asterisks* indicate the significant differences between initial medium and optimized fermentation medium. *P* values were calculated by two-taild Student’s t test. Single asterisk indicates *P* < 0.05

Although nitrogen limitation had been reported to improve the synthesis of PHB by increasing the NADH pool when carbon sources are abundant [34, 35], here, the insufficient supply of sugar in the later period of the exponential phase might be the main factor hindering the increase of the NADH concentration and PHB content (Figs. 2a and 5b). To further increase the yield of FK520 by strengthening PHB metabolism, the addition of carbon sources was optimized as shown in Fig. 6. Firstly, the addition of starch and dextrin in the fermentation medium was optimized by single-factor experiments (Figs. 6a, b). When the addition of starch was increased to 24 g/L, the FK520 yield reached up to 576.14 mg/L, which was 12.64% higher than in the initial fermentation medium (511.50 mg/L) (Fig. 6a). When the addition of dextrin was increased to 56 g/L, the FK520 yield reached up to 602.73 mg/L, 17.84% higher than in the initial fermentation medium (511.50 mg/L) (Fig. 6b). Then, the mixed addition of starch and dextrin was optimized in two-factor experiments (Fig. 6c). When the addition of starch and dextrin was increased to 22 g/L and 52 g/L respectively, the FK520 production reached up to 626.30 mg/L, which was 1.22-fold higher than in the initial medium (511.50 mg/L) and 2.11-fold higher than that of the parent strain FS35 in the initial medium (296.29 mg/L) (Fig. 6c). Thus, 22 g/L starch and 52 g/L dextrin were determined as the optimal concentrations of carbon sources for the strain OphaCfkbU to produce FK520. In the optimal fermentation medium, strain OphaCfkbU exhibited higher concentrations of cofactors, so that it stored more PHB than in the initial medium (Fig. 6d). This indicated that the addition of carbon sources indeed increased the production of FK520 by playing the role of a carbon reservoir to a greater extent.

## Discussion

The supply of precursors from the primary metabolic pathways is crucial for the biosynthesis of secondary metabolites [36]. FK520 is a secondary metabolite produced by *Streptomyces hygroscopicus* var. *ascomyceticus*, so enhancing the supply of precursors is an efficient way to increase the production of this antibiotic [6]. It is well-known that the exogenous feeding of important metabolites is an effective method to increase the biosynthesis of precursors [37–39]. A previous study showed that the exogenous addition of 1.0 g/L crotonic acid increased the production of FK520 in the *fkbS* overexpression strain SFK-OfkbS by 8.75% due to an increased supply of the precursor ethylmalonyl-CoA [32]. Similarly, it was reported that the addition of sodium decanoate as a precursor in the fermentation medium stimulated the production of daptomycin in *Streptomycete roseosporus* NRRL11379 by strengthening the expression of key enzymes in the biosynthetic pathway [27]. Unlike the typical PHB-producing strain *R. eutropha* H16, where PHB is accumulated and then maintained at a high level throughout the fermentation period [40, 41], PHB in *S. hygroscopicus* var. *ascomyceticus* was decomposed during the stationary phase of fermentation to increase the production of FK520 by enhancing the supply of various precursors. Compared with the strategy of exogenous feeding, the manipulation of PHB metabolism seems to be more cost-effective, since the accumulation of PHB requires only cheap carbon sources, such as starch and dextrin, rather than expensive amino acids or other metabolic intermediates. Moreover, these cheap carbon sources only need to be added during the preparation of fermentation medium, but not in the fermentation process, reducing the possibility of bacterial contamination. Furthermore, the decomposition of PHB during the stationary phase not only promoted the biosynthesis of all precursors by increasing the global carbon flux, but also ensured the preferential supply to the specific precursor, eliminating the competitive use of these precursors for by-products synthesis. Therefore, the strategy of using PHB as an intracellular carbon reservoir increased the production of FK520 to a greater extent.

In fact, it was reported that the introduction of PHB synthesis pathway could promote the production of primary metabolites such as L-threonine, succinate and L-arginine, by driving the carbon flux of related primary metabolic pathways [18, 19, 42]. Even so, PHB metabolism was also once thought to be an ineffective cycle in the biosynthesis of dicarboxylic acids. In *Methylobacterium extorquens*, PHB metabolism decreased the yields of mesaconic acid and 2-methylsuccinic acid by competing for the common precursor 3-hydroxybutyryl-CoA with methylsuccinyl-CoA and mesaconyl-CoA in the EMCP. However, although the knockout of PHB synthesis gene *phaC* temporarily increased the yield of these dicarboxylic acids, the growth rate of the mutant strain decreased significantly. Moreover, the knockout strain was genetically unstable and frequently developed suppressor mutants, which grew at the same rate as the wild-type strain but produced less dicarboxylic acids [21]. Similarly, the deletion of the *phaC* gene in *Bacillus thuringiensis* resulted in a low biomass yield and a sporulation-deficient phenotype. Proteomic analysis showed that the basic metabolism was disrupted in the knockout strain, and a variety of futile products were synthesis [43]. Therefore, we did not consider inactivating the PHB synthesis gene in this study, but co-overexpressed the PHB synthesis gene *phaC* and the decomposition gene *fkbU* in the parent strain FS35. The qRT-PCR analysis of *hcd* and *fkbE* verified that the co-overexpression did not caused metabolic disturbance, but instead resulted in more accumulation of PHB during the exponential phase and more decomposition of PHB during the stationary phase to provide additional carbon sources (Additional file 1: Figure S5). All of these findings indicated that PHB metabolism is not a useless cycle, and it plays an indispensable role in maintaining strain growth and regulating intracellular carbon fluxes.

The effect of PHB on the production of primary metabolites is uncertain, because primary metabolites are produced during the synthesis of PHB. This temporal overlap allows PHB to increase the yield of some primary metabolites by driving the metabolic flux, or to reduce the production of other primary metabolites by competing for common precursors, as described above. By contrast, this study showed that the biosynthesis of the secondary metabolite FK520 was accompanied by the decomposition of PHB. When FK520 was synthesized during the later fermentation phase, PHB was decomposed into monomers, providing the carbon moieties for the synthesis of precursors. At this point, the role of PHB metabolism is not as a competitor, but as a contributor to the production of secondary metabolites. This is the first report that the decomposition of PHB can promote the production of secondary metabolites by enhancing the carbon flux of the primary metabolic pathways. A previous paper speculated that PHB might serve as a carbon reserve for the production of antibiotics in some *Streptomyces* species, such as *S. venezuelae* and *S. coelicolor* A3 (2) [44], but there was no detailed evidence to support this idea. Here, we explored the role of PHB as an intracellular carbon reservoir in enhancing the output of FK520 in detail, so as to provide a reference for future research on the effect of PHB on the production of other antibiotics.

In contrast with the defective growth of the knockout strains mentioned above, the co-overexpression strain OphaCfkbU accumulated more biomass along with the biosynthesis of PHB. A previous study also showed that the introduction of the PHB gene cluster could improve the growth of *Corynebacterium crenatum* by promoting the consumption of glucose [19]. However, there were relatively few studies on the PHB in *Streptomyces*, mainly focusing on functional analysis of PHB depolymerase [44–46]. Therefore, the effect of PHB on the growth of *Streptomyces* was unknown. Here, the comparative transcriptomic analysis showed that when carbon sources were abundant in the environment, polyhydroxybutyrate was stored and stimulated the growth of the strain by promoting the utilization of carbon sources. This is a systematic analysis of the mechanism through which PHB accumulation influences the growth of bacteria, providing a reference for future research.

In most PHB-producing bacteria, the synthesis of 3-hydroxybutyryl-CoA is catalyzed by the NADPH-dependent acetoacetyl-CoA reductase (encoding by *phaB*), and the accumulation of PHB is mainly depended on the supply of NADPH [35]. However, NADPH is mainly responsible for reduction in anabolism [47, 48], so the biosynthesis of PHB is often limited by the lack of NADPH. Unlike the shortage of NADPH, NADH cans be synthesized in large quantities via the EMP and TCA when the sufficient carbon sources are available [34]. Previous studies increased the production of PHB by replacing the NADPH-dependent acetoacetyl-CoA reductase with the NADH-dependent acetoacetyl-CoA reductase [33, 34]. In this study, the synthesis of PHB monomer was shown to be catalyzed by an NADH-dependent 3-hydroxybutyryl-CoA dehydrogenase in *Streptomyces hygroscopicus* var. *ascomyceticus*. This is an example in which the accumulation of PHB does not depend on NADPH but on NADH, providing a reference for research on PHB synthesis in other bacteria.

In this study, the optimization of carbon addition increased the production of FK520 by strengthening the PHB metabolism. In general, the addition of excess carbon source in the fermentation medium may inhibit strain growth and the biosynthesis of target products. This problem is usually solved by the optimization of medium composition, fed-batch fermentation or adaptive evolution for tolerance [11, 49, 50]. For example, the production of 16α-hydrohydrocortisone by *Streptomyces roseochromogenes* was increased significantly by using an optimized medium based on malt extract and yeast extract [50, 51]. High addition of glucose and maltose in the fermentation medium caused low cell density and decreased acarbose production in *Streptomyces* M37. Thus, a two-stage fermentation strategy was developed to weaken the inhibition and increase the production of acarbose [52]. Similarly, fed-batch fermentation on glucose resulted in a high cell density and high yield of valinomycin by reducing the glucose inhibition [49]. In a different approach, a glucose-tolerance strain was selected using stepwise UV mutagenesis, achieving a high yield of rapamycin in *Streptomyces hygroscopicus* [11]. Here, PHB was stored when carbon sources are abundant, and depolymerized to monomers for the biosynthesis of FK520 precursors when carbon sources are insufficient. PHB therefore acts as a buffer role to a certain extent, avoiding the inhibition by excessive carbon sources. Therefore, the enhancement of PHB metabolism could be used as a new strategy for high-density culture and high yield of secondary metabolites. It is well known that nitrogen limitation in the presence of sufficient carbon sources can increase the NADH pool and repress the TCA cycle, resulting in more acetyl-CoA flowing into the EMCP for the synthesis of PHB [34, 35]. Consequently, enhancing PHB metabolism by triggering nitrogen limitation will be our next goal.

## Conclusions

Based on systematic transcriptional analysis, we demonstrated for the first time that polyhydroxybutyrate acts as an intracellular carbon reservoir in *S. hygroscopicus* var. *ascomyceticus*. When carbon sources were abundant, polyhydroxybutyrate was stored and stimulated strain growth by promoting the utilization of carbon sources. When carbon sources became insufficient, polyhydroxybutyrate was depolymerized into monomers for the biosynthesis of FK520 precursors. The combination of genetic manipulation and optimized addition of carbon sources eventually increased the yield of FK520 2.11-fold, reaching 626.30 mg/L. To our best knowledge, this is the first report that polyhydroxybutyrate metabolism can promote the synthesis of antibiotics, providing a new strategy for achieving high yields of other secondary metabolites.

## Materials and methods

### Strains, plasmids and growth conditions

*S. hygroscopicus* var. *ascomyceticus* FS35 selected from S. *hygroscopicus* var. *ascomyceticus* ATCC 14891 after femtosecond laser irradiation was used as the parent strain [2]. All the strains and plasmids used in this work are listed in Additional file 1: Table S1. Strain FS35 and its derivatives was cultured and passaged on the MS solid medium (a solid medium for the culture of *Streptomyces*, including 20 g/L soybean cake meal, 20 g/L mannitol and 20 g/L agar powder) at 28 °C. When there were black spores formed on the MS medium, then spores were inoculated into the liquid seed medium and shaken for 60 h at 28 °C and 220 rpm. The composition of seeds medium was same as described previously [6]. And then the seeds cultures were transferred into the fermentation medium with the inoculation of 10%. The fermentation broth were shaken for 192 h at 28 °C and 220 rpm to detect various indexes. The fermentation medium used for the measurement of various fermentation parameters contained 20 g/L soluble starch, 40 g/L dextrin, 5 g/L yeast powder, 5 g/L peptone, 5 g/L corn steep liquor, 1 g/L K_2_HPO_4_, 1.5 g/L (NH_4_)_2_SO_4_, 0.5 g/L MnSO_4_, 1 g/L MgSO_4_·7H_2_O, 1 g/L CaCO_3_, and 2.5 mL/L soybean oil. The optimum concentration of carbon sources suitable for the mutant strain was presented in the results section. *Escherichia coli* DH5α was used for plasmid construction. *Escherichia coli* ET12567 (pUZ8002) was used as the donor strain for intergeneric conjugation with S. *hygroscopicus* var. *ascomyceticus*. *E. coli* strains were cultured in Luria–Bertani (LB) medium at 37 °C. The plasmid pIB139, which contains the strong promoter ermE*p, was used to overexpress genes in strain FS35. The plasmid pBHR68, containing the PHB synthesis operon (*phaABC)* from *R. eutropha* H16, was kindly gifted from Professor Tao Chen (Tianjin University, China).

### Construction of overexpression strain

To obtain the co-overexpression strain OphaCfkbU, the complete *phaC* gene was amplified from the plasmid pBHR68 using the primers OphaC-fkbU-F1/OphaC-fkbU-R1, and the complete *fkbU* gene was amplified from the genome of FS35 using the primers OphaC-fkbU-F2 /OphaC-fkbU-R2. All primers used for gene manipulations are listed in Additional file 1: Table S2. Then, the two fragments were fused by overlap-extension PCR using the primers OphaC-fkbU-F1/OphaC-fkbU-R2. The fusion fragment was inserted into the vector pIB139 between the NdeI and XbaI sites to construct the co-overexpression vector pIBOPF, in which the fusion fragment was under the strong constitutive promoter ermE*p. The recombinant plasmid pIBOPF was transformed into competent cell of *E. coli* ET12567 (pUZ8002) to obtain transformant. The transformant was cultured in liquid LB medium containing 50 μg/mL apramycin sulfate, 25 μg/mL kanamycin and 25 μg/mL chloramphenicol. When the OD value reached 0.4–0.6, the transformant was mixed with fresh spores of strain FS35 for conjugal transfer. Conjugal transfer was performed according to standard protocols [7]. Positive single colonies, which contained the co-overexpression vector pIBOPF, were selected from MS solid medium containing 50 μg/mL nalidixic caid and 50 μg/mL apramycin sulfate. Finally, the positive single colonies were relaxed cultured on MS solid medium without antibiotics to obtain co-overexpression strain OphaCfkbU. To obtain the overexpression strain OfkbU, the complete *fkbU* gene was amplified from the genome of FS35 using the primers OfkbU-F/OfkbU-R and inserted into the vector pIB139 in the way described above to obtain the overexpression plasmid pIBOF. Then the overexpression plasmid pIBOF was introduced into FS35 by conjugal transfer to obtain the overexpression strain OfkbU.

### Determination of FK520, FK523, biomass and PHB content

For the measurement of the yield of FK520 and FK523, 2 mL of fermentation broth was mixed with 3 mL of ethanol and was extracted by ultrasound for 30 min. Then the mixture was centrifuged for 10 min at 8000×*g*. The supernatant was filtered by a 0.22 μm of oily filter and quantified by liquid chromatography on a 1100 series instrument (Agilent, USA), equipped with a C-18 column (150 mm × 4.6 mm, 3.5 μm; Agilent). The column temperature was 60 °C and the detection wavelength was set to 205 nm. The mobile phase and the gradient elution method was same as described previously [32]. The flow rate was 2 mL/min, and the injection volume was 20 μL.

To obtain the biomass concentration, 5 ml of fermentation broth was washed once with 0.1 M-HCl solution and twice with Milli-Q water. After centrifugation for 10 min at 8000 × *g*, the wet cell pellet was dried in oven at 80 °C until constant weight to measure the dry cell weight (DCW). The biomass concentration is the ratio of DCW to the volume of fermentation broth. The residual total sugars in fermentation broth were quantified in the same way as described previously [53].

To measure the intracellular content of PHB, the fermentation broth was centrifuged for 20 min at 8000 × *g* and washed twice with deionized water. Wet hyphae was freeze-dried (Christ ALPHA 1–2 LD plus, Germany) and 60 mg of the lyophilized mycelium was put into an airtight tube with 2 mL of esterification reagent and 2 mL of chloroform. The esterification reagent was contained 0.1 g of benzoic acid (internal standard), 3 mL of concentrated sulfuric acid and 97 mL of methyl alcohol. The esterification reaction was carried out in a calorstat for 4 h at 100 °C. Then the mixture were cooled to room temperature, and combined with 1 mL of deionized water. The chloroform phase in the lower layer was collected and quantified by gas chromatography (GC) on a 430-GC instrument (Bruker, Germany) equipped with a BR-5 capillary column (30 m, 0.32 mm, 0.25 μm; Bruker) according to a published method [54]. The detector for GC was flame ion detector (FID) and the temperature of detector was 250 °C. The heating program was 80 °C for 1.5 min, then heated up to 140 °C at a rate of 30 °C /min, and then heated up to 250 °C at a rate of 40 °C /min, finally held at 250 °C for 5 min. The gradient poly-3-hydroxybutyric acid was treated with the above esterification procedure to build the standard curve.

### ***Quantification of NADH, NAD***^+^***, NADPH and NADP***^+^

For the measurement of intracellular concentrations of NADH, NAD^+^, NADPH and NADP^+^, the samples was harvested at 96 h by centrifugation under 8000 × *g* for 10 min. The wet hyphae were rapidly frozen in liquid nitrogen. After washing twice with ice-cold PBS, the samples was mixed with 100 µL of extraction buffer and heat extracted at 60 °C for 5 min. Then the extract was neutralized by 20 µL of assay buffer and 100 µL of the opposite extraction buffer. After centrifugation under 8000 × *g* for 10 min, the supernatant was collected for assays. The whole operation was carried out in strict accordance with the protocols of EnzyChrom™ NAD/NADH Assay Kit (Bioassay Systems, USA). 40 µL of each sample was mixed with 80 µL of working reagent, which was freshly prepared according to the manufacturer’s instructions. After a 15 min of incubation at room temperature, the concentrations of NADH, NAD^+^, NADPH and NADP^+^ were measured by the optical density at 565 nm.

### RNA extraction and cDNA library construction

To record the changes in transcriptome data caused by the co-overexpression, the fermentation broth of FS35 and OphaCfkbU was collected at 50 h. After centrifugation at 8000 × *g* for 10 min, the wet hyphae were rapidly frozen in liquid nitrogen. The total RNA was extracted from the frozen hyphae with Trizol reagent (Invitrogen, USA). The rRNA was removed with Ribo Zero rRNA Removal Kit (Epicentre, USA). RNA degradation and contamination was monitored on 1% agarose gels. RNA purity was checked with the NanoPhotometer ® spectrophotometer (IMPLEN, CA, USA). RNA concentration was measured with Qubit® RNA Assay Kit in Qubit® 2.0 Flurometer (Life Technologies, CA, USA). RNA integrity was assessed with RNA Nano 6000 Assay Kit of the Bioanalyzer 2100 system (Agilent Technologies, CA, USA). The cDNA libraries were generated with NEBNext® UltraTM Directional RNA Library Prep Kit for Ιllumina® (NEB, USA). The library fragments were purified with AMPure XP system (Beckman Coulter, Beverly, USA). After terminal repair and PCR amplification, the quality of the cDNA libraries was assessed on the Agilent Bioanalyzer 2100 systerm.

### Sequencing and data analysis

To analyze the changes in transcriptome data caused by genetic manipulation, the cDNA libraries were clustered on a cBot Cluster Generation System with TruSeq PE Cluster Kit v3-cBot-HS (Ιllumina) according to the manufacturer’s instructions, and sequenced on a Ιllumina HiSeq platform to generate the paired-end reads. The clean reads were obtained by removing the reads containing adapter, reads containing poly-N and low quality reads from raw reads of fastq format. Then they were aligned to the sequenced genome of *S. hygroscopicus* var. *ascomyceticus* FS35 using Bowtie 2–2.2.3 [55]. The numbers of reads mapped to each gene were counted by HTSeq v0.6.1. In order to estimate the expression level of each gene, Fragments Per Kilobase of transcript sequence per Millions base pairs sequenced (FPKM) were calculated based on the length and reads count of mapped genes. After reads counts was adjusted with edger program package through one scaling normalized factor, differential expression analysis was performed using the DEGSeq R package (1.20.0) [56]. A corrected P-value of 0.005 and log_2_ (fold change) of 1 were set as the thresholds for significantly differential expression analysis. GO enrichment analysis of the differentially expressed genes was implemented with GOseq R package [57]. The statistical enrichment of differentially expressed genes into Kyoto encyclopedia of genes and genomes (KEGG) pathways was tested on the KEGG Orthology Based Annotation System (KOBAS) [58]. The raw transcriptomic data and genomic sequences had been uploaded to the Gene Expression Omnibus (GEO) database in National Center for Biotechnology Information (NCBI) (accession number: GSE 143832).

### Quantitative real-time PCR (qRT-PCR) analysis

To verify the different pattern of PHB metabolism during the exponential phase and stationary phase, the transcriptional levels of gene *hcd* and *fkbE* were measured by qRT-PCR at 50 h and 112 h. And to explore the influence mechanism of PHB degradation on FK520 production, the transcriptional levels of the selected genes were measured by qRT-PCR at 112 h. All primers were listed in Additional file 1: Tables S3 and S4. The sample collection method was the same as described above. The total RNA was extracted from frozen hyphae with RNAprep Pure Cell/Bacteria Kit (Tiangen, Beijing, China). The RNA sample was reversely transcribed into cDNA with PrimeScript™ RT reagent Kit (Takara, Japan). The integrity and concentration of RNA sample was detected by 1% agarose gel electrophoresis. Then the RNA sample was reversely transcribed into cDNA by using PrimeScript™ RT reagent Kit with gDNA Eraser (takara, Japan). With the cDNA as template, qRT-PCR was carried out on a LightCycler® 480 using SYBR Green Master Mix (Roche, Switzerland). To exclude DNA contamination, the RNA sample which treated by gDNA Eraser but not reverse transcription was used as a template for negative control. The 16S rRNA was used as the internal reference gene, and the change folds of the selected genes were quantified relatively with comparative *C*_T_ method [36].

To verify the accuracy of transcriptome data, several genes were selected for qRT-PCR analysis. All primers used for accuracy verification of transcriptome data were listed in Additional file 1: Table S5. The procedure was the same as description above. As shown in Additional file 1: Figure S6, the high correlation (r^2^ = 0.9637) between the qRT-PCR validation data and transcriptome data proved the reliability of transcriptomic results.

### Statistical analysis

In this study, the samples for the measurement of fermentation parameters were all taken in five independent biological replicates. The samples for quantitative real-time PCR were all taken in three independent biological replicates. All data were presented as the mean values of respective independent biological replicates and the error bars indicate the standard deviations (SD).

## Supplementary Information

**Additional file 1: Table S1.** Strains and plasmids used in this study. **Table S2.** Primers used for genetic manipulation. **Table S3.** Primers used for transcriptional level analysis of primary metabolism. **Table S4.** Primers used for transcriptional level analysis of ascomycin gene cluster. **Table S5.** Primers used for the verification of transcriptome data. **Figure S1.** Influence of the overexpression of *fkbU* gene on the yield of ascomycin. **Figure S2.** Effect of the co-overexpression on the growth of mycelium in the fermentation broth and solid plates. **Figure S3.** Volcano map of differentially expressed genes caused by the co-overexpression. **Figure S4.** Sequence homology alignment of 3-hydroxybutyryl-CoA dehydrogenase in *Streptomyces hygroscopicus* var. *ascomyceticus* and several other *Streptomyces*. **Figure S5.** Different metabolic patterns of Polyhydroxybutyrate during the exponential phase and the stationary phase. **Figure S6.** Correlation coefficients between the transcriptomic data and qRT-PCR validation data.

## Data Availability

The dataset related to transcriptomic and genome sequences is available in the GEO database, [Accession Number: GSE 143832, https://www.ncbi.nlm.nih.gov/geo/query/acc.cgi?acc=GSE143832]. And the data generated and analyzed during this study are included in the article and its Additional file 1.

## Abbreviations

NADH: Nicotinamide adenine dinucleotide (reduction state)
NAD^+^: Nicotinamide adenine dinucleotide (oxidation state)
NADPH: Nicotinamide adenine dinucleotide phosphate (reduction state)
NADP^+^: Nicotinamide adenine dinucleotide phosphate (oxidation state)
FK520: Ascomycin
FK523: A structural analogue of ascomycin
PHB: Polyhydroxybutyrate
EMCP: Ethylmalonyl-CoA pathway
EMP: Glycolysis pathway
PPP: Pentose phosphate pathway
TCA: Citrate cycle
DCW: Dry cell weight
LB: Luria–Bertani
GC: Gas chromatography
GO: Gene ontology
KEGG: Kyoto encyclopedia of genes and genomes
KOBAS: KEGG Orthology Based Annotation System
NCBI: National Center for Biotechnology Information
GEO: Gene Expression Omnibus
qRT-PCR: Quantitative real-time PCR
ACP: Acyl-carrier protein
PAPS: 3′-Phosphoadenosine 5′-phosphosulfate
DHCHC: 4,5-Dihydroxycyclohex-1-enecarboxylic acid
SD: Standard deviations
FPKM: Fragments Per Kilobase of transcript sequence per Millions base pairs sequenced
FID: Flame ion detector
Lys: Lysine
Val: Valine
MS: A solid medium for the culture of *Streptomyces*
