# Supplementary material for: Enhanced ascomycin production in Streptomyces hygroscopicus var. ascomyceticus by employing polyhydroxybutyrate as an intracellular carbon reservoir and optimizing carbon addition
Source: Microb Cell Fact. 2021 Mar 17;20:70. doi: 10.1186/s12934-021-01561-y (PMC7968196; doi:10.1186/s12934-021-01561-y)
Supplement: Supplementary file 1 — Additional file 1: Table S1. Strains and plasmids used in this study. Table S2. Primers used for genetic manipulation. Table S3. Primers used for transcriptional level analysis of primary metabolism. Table S4. Primers used for transcriptional level analysis of ascomycin gene cluster. Table S5. Primers used for the verification of transcriptome data. Figure S1. Influence of the overexpression of fkbU gene on the yield of ascomycin. Figure S2. Effect of the co-overexpression on the growth of mycelium in the fermentation broth and solid plates. Figure S3. Volcano map of differentially expressed genes caused by the co-overexpression. Figure S4. Sequence homology alignment of 3-hydroxybutyryl-CoA dehydrogenase in Streptomyces hygroscopicus var. ascomyceticus and several other Streptomyces. Figure S5. Different metabolic patterns of Polyhydroxybutyrate during the exponential phase and the stationary phase. Figure S6. Correlation coefficients between the transcriptomic data and qRT-PCR validation data. [file 12934_2021_1561_MOESM1_ESM.docx]

**Additional file 1**

**Microbial Cell Factories**

**Enhanced ascomycin production in *Streptomyces hygroscopicus* var. *ascomyceticus* by employing polyhydroxybutyrate as an intracellular carbon reservoir and optimizing carbon addition**

Pan Wang^1,2^, Ying Yin^1,2^, Xin Wang^1,2^ , Jianping Wen^1,2,*^

^1^ Key Laboratory of Systems Bioengineering (Ministry of Education), Tianjin University, Tianjin, China

^2^ SynBio Research Platform, Collaborative Innovation Center of Chemical Science and Engineering (Tianjin), School of Chemical Engineering and Technology, Tianjin University, Tianjin, China

* Correspondence author: Jianping wen,

Telephone: +86-022-27892061;

Fax: +86-022-27892061;

E-mail: jpwen@tju.edu.cn

**Table S1** Strains and plasmids used in this study

| **Strains/plasmids** | **Descriptions** | **source** |
| --- | --- | --- |
| *S. hygroscopicus* var. *ascomyceticus* | | |
| FS35 | Mutant derived from ATCC 14891 | Lab collection |
| OfkbU | FS35 transformed with pIBOF | This study |
| OphaCfkbU | FS35 transformed with pIBOPF | This study |
| *E.coli* | | |
| DH5α | Plasmid construction and general cloning | Lab collection |
| ET12567/pUZ8002 | Plasmid donor for intergeneric conjugation, Cm^R^, Kan^R^ | Lab collection |
| Plasmids | | |
| pBHR68 | plasmid containing operon *phaABC* | Lab collection |
| pIB139 | Integrative shuttle plasmid containing *ermE*p* | Lab collection |
| pIBOF | pIB139 based integrative plasmid containing *fkbU* gene, Apr^R^ | This study |
| pIBOPF | pIB139 based integrative plasmid containing *fkbU* gene and *phaC* gene, Apr^R^ | This study |

**Table S2** Primers used for genetic manipulation

| **Primers** | **Sequence (5’-3’)** |
| --- | --- |
| OfkbU-F | GGAATTCCATATGCAGCCGCCGCCGTTCCGGGGAA |
| OfkbU-R | TGCTCTAGATCAGGGACAGGTGTCGTTCGCGACGGTGAAG |
| OphaC-fkbU-F1 | GGAATTCCATATGGCGACCGGCAAAGGCGCGGCAG |
| OphaC-fkbU-R1 | GCGGCTGCATTGGATCCTTCATGCCTTGGCTTTGACGTATC |
| OphaC-fkbU-F2 | CAAGGCATGAAGGATCCAATGCAGCCGCCGCCGTTCC |
| OphaC-fkbU-R2 | CTAGTCTAGATCAGGGACAGGTGTCGTTCGCGACG |

Underlined letters represent restriction sites.

**Table S3** Primers used for transcriptional level analysis of primary metabolism

| **Genes** | **Primers (5’-3’)** |
| --- | --- |
| *pfk* | ACCGCCGAATCGCATCAG |
|  | GCTCCTCGACCATCTTCACCA |
| *glpX* | TCGGGGAAGGGGAGAAGG |
|  | AGCACGGAGACGGCGTTG |
| *gapA* | GTCACGCTCGCCTACGGT |
|  | AGGTCGTCCAGCACGGAC |
| *neo* | CCCGCTCTTCCGTTACCTC |
|  | AACTCCTGGATGTCCACGTTG |
| *pyk* | GTACGAGCAGATCAAGGCACT |
|  | TCTCCAGCGAGGCTTTGC |
| *pdhA* | AGGAGGCGGCGCAGATC |
|  | GGTGGAAGTTGTTGCTGTTGG |
| *atoB* | TCCACCGAGAAGCACAACAC |
|  | GACCACAGGGTCGCCCTT |
| *pkcA* | CCAAGTACATCTCGCACTTCC |
|  | TGAGCTTGAGGATCAGCATGT |
| *gltA* | AGGGGAAGCCGATGGTGC |
|  | ACACGGGCGGTGAAGGTG |
| *fumC* | GGCGTGGTGGACAAGGAC |
|  | CCTCGTTGGTGTTCATGTTGG |
| *korB* | CGTCCAGGGGCTGACCGT |
|  | GATGCGCACCTTGGGCAC |
| *idh* | GCGACGTACTCGTTCCTGC |
|  | CGAGGTACTCCGGGAACTG |
| *aceB* | CTCCACCACCGCTTCACC |
|  | GGTCTCCGGCAGGAAGTCC |
| *mut* | CAGCAGCCGTACAACAACG |
|  | CGGTCTCCTCCATGAGCAC |
| *bccA* | TGCTTCATGGAGATGAACACC |
|  | CGGTCTCGGCACAGATACG |
| *ecm* | GGCTGTCGGTCGCCTTC |
|  | CGCGGTCGCGTTGATG |
| *hcd* | GCGATCTGGGCAAGGAGC |
|  | CCCGCAGCCGAGTCTCAT |

**Table S4** Primers used for transcriptional level analysis of ascomycin gene cluster

| **Genes** | **Primers (5’-3’)** |
| --- | --- |
| *fkbW* | CACTGCGCCCGTCTGTCC |
|  | TCAGCCCTGCCCAGTCCG |
| *fkbU* | GACCGCTATGGCTTCCTGC |
|  | GGTGATGTAGGTGCGCTGG |
| *FkbR2* | CGCACGGTCCTGGAGACC |
|  | TGTCGGTGACGCTCTGCC |
| *FkbR1* | CGGCTGGAGCGACGAGT |
|  | GTGTCGGGGTCGATCAGG |
| *fkbE* | CCGGTCTGCTCGTGGTTTC |
|  | CGGTTCAGCCAGACGAAGTG |
| *fkbF* | GTGGTCGCACTCGACCGA |
|  | CAGTCCACCGTGCCGTTG |
| *fkbG* | TACGGAAGGTGTCCCTGCG |
|  | CCGGTGTACGTCCCGATCT |
| *fkbH* | GGGACCTGGACAACACCTTG |
|  | CGTTCCAGACGCTCCCAC |
| *fkbI* | CGGGAGAACGGCGAGTTC |
|  | CCGCTGGTCAGCTCCTTCA |
| *fkbJ* | GACCGCACCCGTCAAGGA |
|  | CGAACAGCGACGTGAGGC |
| *fkbK* | GGATCGCCACCGAACTGC |
|  | CGAGTACCTCCGCCTTGACC |
| *fkbL* | CGTGACATCAAGCGGATTCTC |
|  | GGCATGAACTCGATGACGC |
| *fkbC* | CGTGCGGTGTTGTTGGGC |
|  | CAGAACACGCCATCAACTCCA |
| *fkbB* | ACGCCGAACTCGCCCACT |
|  | GAGGTCCTCGTAGGAGACGCA |
| *fkbO* | CGCCGTATTGCCGCTTC |
|  | CGTGCCAGACCTCGGTGAA |
| *fkbP* | TGATCCGCCTCGGTGACG |
|  | TGGGCTGCGAGTTCATGTTG |
| *fkbA* | GCCCTGGAGACCTCGTGG |
|  | ATGCCGTACCCCTGGAAGA |
| *fkbD* | AGAGCGGCACGGTGGGTT |
|  | CGGTTGTGCTCCGGTGAGTC |
| *fkbM* | TTGCCGAACGGGACGACG |
|  | ACACCAGGACACTCCAGATGC |
| *fkbN* | CTGCGCCACTACCAGGAGAT |
|  | GGTCCGTGTCCGTGCCTC |
| *fkbQ* | CGCACCTGATCGTCTCGG |
|  | CGTAGTAGTCCGCCCGCAAC |
| *fkbS* | GGGATACGAGCACGCCTTTG |
|  | AGCGGGTAGGTCACCGACAG |

**Table S5** Primers used for the verification of transcriptome data

| **Genes** | **Primers (5’-3’)** |
| --- | --- |
| 1_7327 | ATGGCCCTGTCGTACCTGC |
|  | CCTCGTACGGACGGTGCA |
| 1_4423 | TCGGGGAAGGGGAGAAGG |
|  | GTATCGACGACCCCAAGACG |
| 1_6088 | TCAGCTGGAGGCCGTGGA |
|  | GATGAACCGCCGCTTGG |
| 1_2437 | AGCTCCCGCCGCATCAC |
|  | CAGTTGCCCATCGAGTCGC |
| 1_2420 | CCCGTCGTGGTCGTCATC |
|  | CTGGTCCTCCAGGATGGC |
| 1_2422 | ACCAGCACCCGTGGTTCC |
|  | CCGACTCCCAGTCGTTCG |
| 1_5981 | ACACCGAACGCCATGTGC |
|  | CGCCCGTCTCGTCATTGG |
| 1_6695 | GCCGTGAGGACTCCGAGAC |
|  | GGGCTGGACGTCGCAGTAG |
| 1_5180 | GCACCGCCAACACCTTCA |
|  | GGAGCGGACCAGCACGAT |
| 1_4132 | CTCGACACCGATCATCAACCAC |
|  | TGGGCTGCTCCACGCAAT |
| 1_6817 | CCCCAGAGCGTCGGAACA |
|  | CCCCATTCGGTGAAGTAGCC |
| 1_3191 | CACCTCCGTCATCAAACCG |
|  | GCTCGTCCTTGACCTCGTG |

**Figure S1**


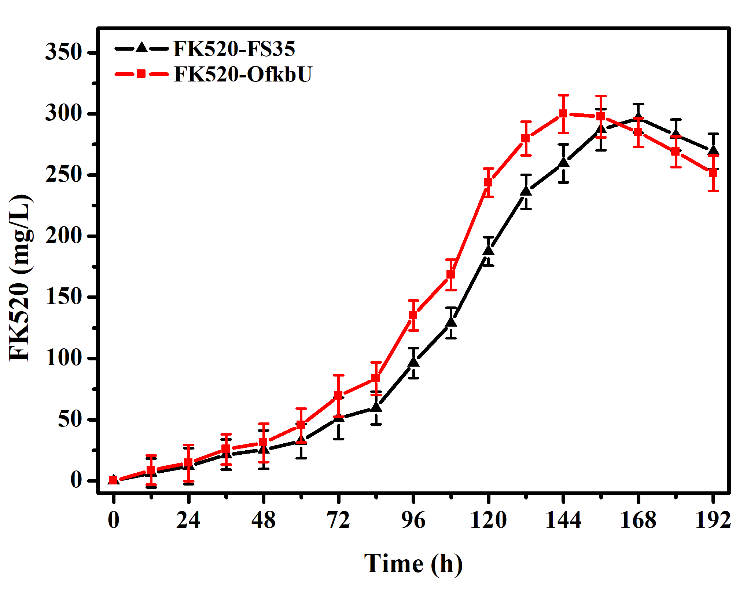


**Figure S1** Influence of the overexpression of *fkbU* gene on the yield of ascomycin. The data represent the mean values of five independent biological replicates, and the error bars represent the standard deviations.

**Figure S2**

**
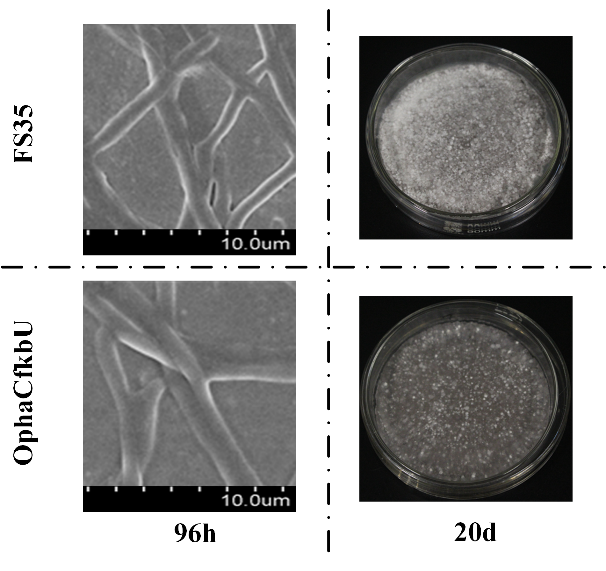
**

**Figure S2** Effect of the co-overexpression on the growth of mycelium in the fermentation broth and solid plates. The mycelium in the fermentation broth was photographed under the scanning electron microscopy, and the scale shows the magnification.

**Figure S3**

**
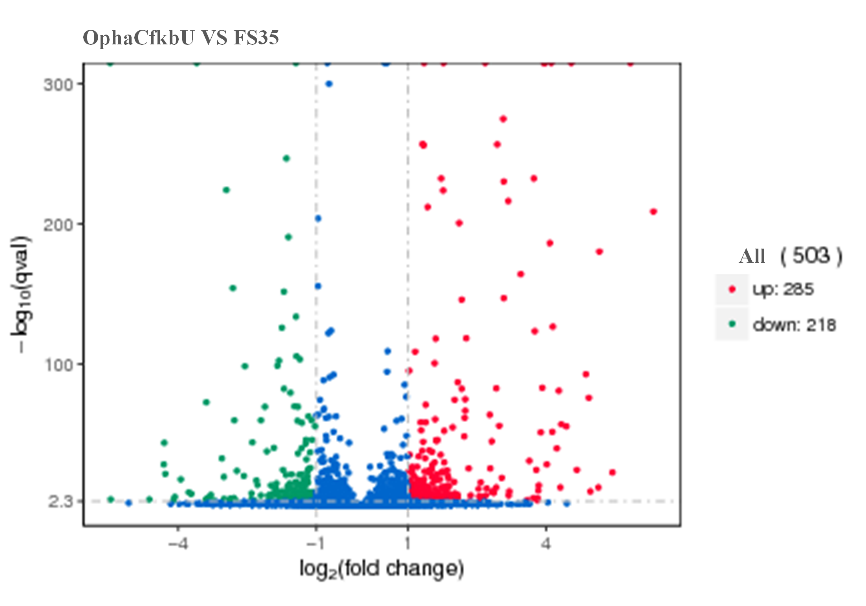
**

**Figure S3** Volcano map of differentially expressed genes caused by the co-overexpression. The The X-axes represents the logarithmic change folds of differential expression. The Y-axes represents the statistical significance of differential expression. Red dots represent the genes whose expression is significantly up-regulated. Green dots represent the genes whose expression is significantly down-regulated. Blue dots represent the genes with no significant difference in expression.

**Figure S4**


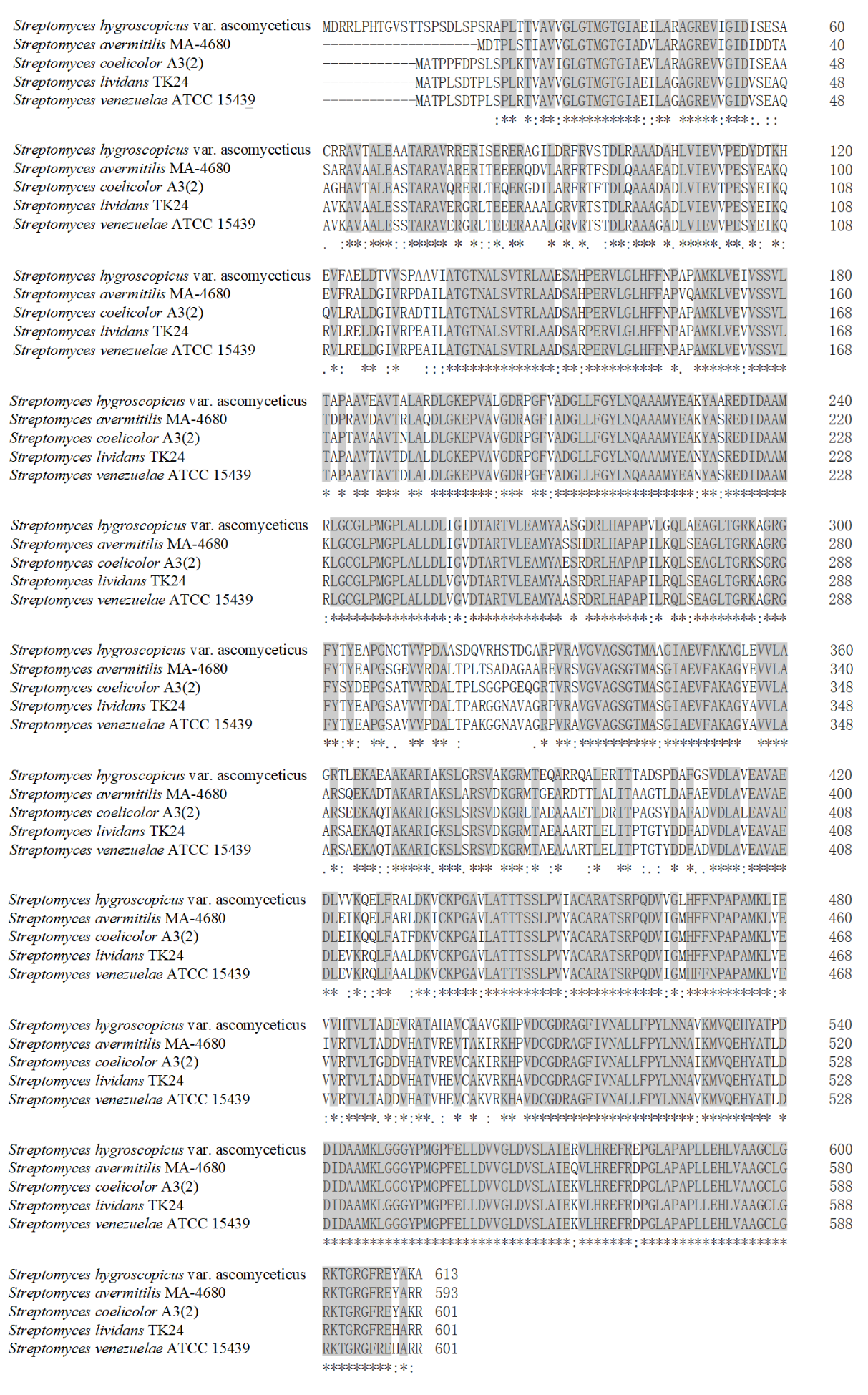


**Figure S4** Sequence homology alignment of 3-hydroxybutyryl-CoA dehydrogenase in *Streptomyces hygroscopicus* var. *ascomyceticus* and several other *Streptomyces*. The shaded boxes and asterisks represent the exactly same sequences in these five strains.

**Figure S5**

**
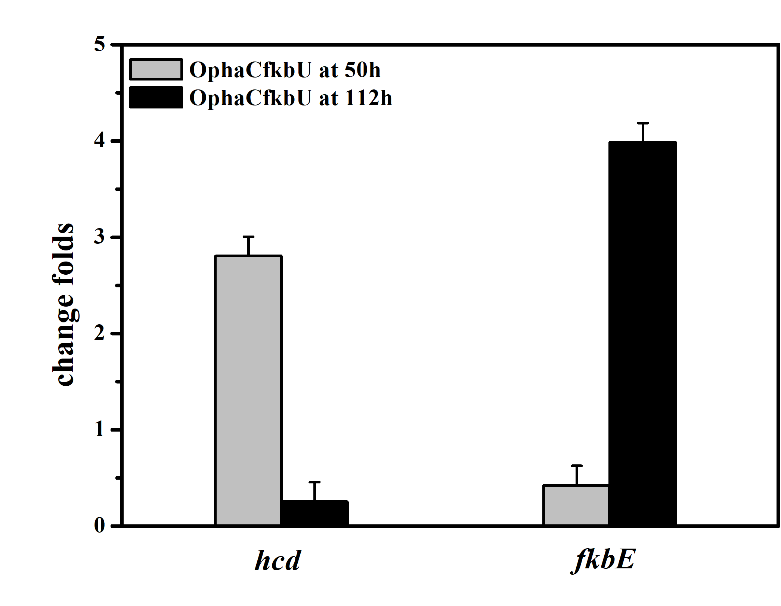
**

**Figure S5** Different metabolic patterns of Polyhydroxybutyrate during the exponential phase and the stationary phase. The transcriptional levels of the genes in the strain FS35 were defined as 1 and used as the control. The data represent the mean values of three independent biological replicates, and the error bars represent the standard deviations.

**Figure S6**


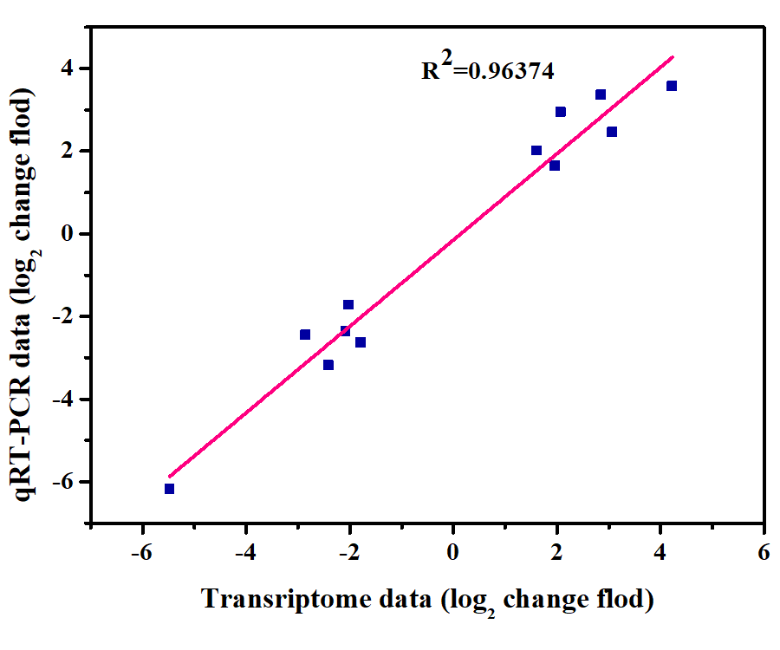


**Figure S6** Correlation coefficients between the transcriptomic data and qRT-PCR validation data. The values in coordinates represent the logarithmic change folds of differentially expression between strain FS35 and OphaCfkbU. The qRT-PCR validation data represent the mean values of three independent biological replicates.
